# Author Correction: Rhinovirus-induced epithelial RIG-I inflammasome suppresses antiviral immunity and promotes inflammation in asthma and COVID-19

**DOI:** 10.1038/s41467-023-39275-x

**Published:** 2023-06-13

**Authors:** Urszula Radzikowska, Andrzej Eljaszewicz, Ge Tan, Nino Stocker, Anja Heider, Patrick Westermann, Silvio Steiner, Anita Dreher, Paulina Wawrzyniak, Beate Rückert, Juan Rodriguez-Coira, Damir Zhakparov, Mengting Huang, Bogdan Jakiela, Marek Sanak, Marcin Moniuszko, Liam O’Mahony, Marek Jutel, Tatiana Kebadze, David J. Jackson, Michael R. Edwards, Volker Thiel, Sebastian L. Johnston, Cezmi A. Akdis, Milena Sokolowska

**Affiliations:** 1 Swiss Institute of Allergy and Asthma Research (SIAF), University of Zurich, Herman-Burchard-Strasse 9, 7265 Davos Wolfgang, Switzerland; 2 Christine Kühne – Center for Allergy Research and Education (CK-CARE), Herman-Burchard-Strasse 1, 7265 Davos Wolfgang, Switzerland; 3 Department of Regenerative Medicine and Immune Regulation, Medical University of Bialystok, Waszyngtona 13 Str., 15-269 Bialystok, Poland; 4 Functional Genomics Center Zurich, ETH Zurich/University of Zurich, Winterthurerstrasse 190, 8057 Zurich, Switzerland; 5 Institute of Virology and Immunology (IVI), Laenggassstrasse 122, 3012 Bern, Switzerland; 6 Department of Infectious Diseases and Pathobiology, Vetsuisse Faculty, University of Bern, Laenggassstrasse 122, 3012 Bern, Switzerland; 7 Graduate School for Cellular and Biomedical Sciences, University of Bern, Mittelstrasse 43, 3012 Bern, Switzerland; 8 Division of Clinical Chemistry and Biochemistry, University Children’s Hospital Zurich, Raemistrasse 100, 8091 Zurich, Switzerland; 9 Children’s Research Center, University Children’s Hospital Zurich, Raemistrasse 100, 8091 Zurich, Switzerland; 10 IMMA, Department of Basic Medical Sciences, Facultad de Medicina, Universidad San Pablo-CEU, CEU Universities Madrid, C. de Julian Romea 23, 28003 Madrid, Spain; 11 Centre for Metabolomics and Bioanalysis (CEMBIO), Department of Chemistry and Biochemistry, Facultad de Farmacia, Universidad San Pablo-CEU, CEU Universities Madrid, Urb. Monteprincipe 28925, Alcorcon, Madrid Spain; 12 Department of Internal Medicine, Jagiellonian University Medical College, M. Skawinska 8 Str., 31-066 Krakow, Poland; 13 Department of Allergology and Internal Medicine, Medical University of Bialystok, M. Sklodowskiej-Curie 24A Str., 15-276 Bialystok, Poland; 14 Department of Medicine and School of Microbiology, APC Microbiome Ireland, University College Cork, College Rd, T12 E138 Cork, Ireland; 15 Department of Clinical Immunology, Wroclaw Medical University, wyb. Lidwika Pasteura 1 Str, 50-367 Wroclaw, Poland; 16 ALL-MED Medical Research Institute, Gen. Jozefa Hallera 95 Str., 53-201 Wroclaw, Poland; 17 National Heart and Lung Institute, Imperial College London, Guy Scadding Building, Cale Street, London, SW3 6LY UK; 18 Department of Infectious Diseases, Imperial College London, School of Medicine, St Mary’s Hospital, Praed Street, London, W21NY UK; 19 Guy’s Severe Asthma Centre, School of Immunology & Microbial Sciences, King’s College London, Strand, London, WC2R 2LS UK; 20 Guy’s & St Thomas’ NHS Trust, St Thomas‘ Hospital, Westminster Bridge Rd, London, SE1 7EH UK; 21 Asthma UK Centre in Allergic Mechanisms of Asthma, Norfolk Place, London, W2 1PG UK; 22 Multidisciplinary Center for Infectious Diseases, University of Bern, Hallerstrasse 6, 3012 Bern, Switzerland; 23 Imperial College Healthcare HNS Trust, The Bays, S Wharf Rd, London, W2 1NY UK

**Keywords:** Viral infection, Asthma, RIG-I-like receptors, Mucosal immunology, Infection

Correction to: *Nature Communications* 10.1038/s41467-023-37470-4, published online 22 April 2023

In this article the author name David J. Jackson was incorrectly written as J. David Jackson, the author name Michael R. Edwards was incorrectly written as R. Michael Edwards, the author name Sebastian L. Johnston was incorrectly written as L. Sebastian Johnston and the author name Cezmi A. Akdis was incorrectly written as A. Cezmi Akdis. The original article has been corrected.

